# Demographic and Behavioral Profiles of Nonbinary and Binary Transgender Youth

**DOI:** 10.1089/trgh.2018.0068

**Published:** 2019-10-21

**Authors:** Kieran Todd, Sarah M. Peitzmeier, Shanna K. Kattari, Michael Miller-Perusse, Akshay Sharma, Rob Stephenson

**Affiliations:** ^1^ Center for Sexuality and Health Disparities, University of Michigan School of Nursing, Ann Arbor, Michigan.; ^2^ Department of Health Behavior and Biological Sciences, University of Michigan School of Nursing, Ann Arbor, Michigan.; ^3^ School of Social Work, University of Michigan, Ann Arbor, Michigan.; ^4^ Department of Systems, Population and Leadership, University of Michigan School of Nursing, Ann Arbor, Michigan.

**Keywords:** gender identity, HIV testing, nonbinary, online survey, transgender youth

## Abstract

**Purpose:** Emerging literature suggests there may be important differences in the demographic characteristics and health profiles of nonbinary transgender youth compared to binary transgender youth.

**Methods:** Between June 2017 and June 2018, 202 transgender youth aged 15–24 years were recruited into a randomized trial of home HIV testing, Project Moxie. This analysis compares demographic and health risk behavior characteristics between youth reporting nonbinary and binary transgender identities in baseline surveys.

**Results:** Nonbinary youth were significantly less likely to have accessed medical interventions to affirm their gender than binary youth (8.4% vs. 46.2%), and less likely to be living currently as the gender that most affirms them (80.7% vs. 91.6%). While there were no significant differences in the low levels of resilience reported across the sample, nonbinary youth reported significantly higher levels of stress. Health risk behaviors were generally high across nonbinary and binary participants, with no significant differences in sexual partner count, condomless sex, alcohol use, tobacco, marijuana, or other drug use.

**Conclusion:** Findings affirmed many similarities, and key disparities, between nonbinary and binary transgender youth. Research and interventions dedicated to the unique needs and experiences of nonbinary transgender youth to address high levels of health risk behaviors and stress are critical.

## Introduction

Transgender describes those whose sex assigned at birth does not align, according to societal expectations, with their gender identity. Although there has been a significant growth of research that has focused on the health of transgender individuals, this has largely been explored within binary categories of gender (man/woman or trans man/trans woman), failing to capture the unique experiences of nonbinary individuals.^1,2^ Nonbinary describes individuals whose identity is not exclusively man or woman. While some nonbinary individuals identify as both men and women, others have identities that are on the spectrum between man and woman, a different gender entirely, or do not identify with any gender.^3,4^

These individuals also may or may not identify as neither transgender nor cisgender. Few studies explicitly include nonbinary individuals when recruiting transgender populations, often taking the approach of categorizing participants as either transgender men or transgender women, not providing an opportunity for participants to report a nonbinary gender.^5–7^ This approach limits understanding of potential differences between binary and nonbinary populations, and does not include the lived experiences of nonbinary individuals.^8–13^ This is a critical knowledge gap as nonbinary individuals likely represent a large and growing percentage of the transgender community: The 2015 United States Transgender Survey included 27,715 respondents, of whom 35% reported a nonbinary gender identity.^7^

The dearth of information about the demographic and health of nonbinary individuals has limited understanding of this population's specific needs, and hampered the development of programming and interventions accurately reflecting their needs and realities. This is especially relevant when working with nonbinary adolescents. Adolescence is a time of identity discovery and evolution, and while there is an increasing attention to the needs and behaviors of transgender youth, there is a lack of knowledge detailing the unique needs of nonbinary youth.^1,14–17^

Adolescence is also a critical developmental period where health disparities may emerge. The small amount of research that has contrasted the mental health of binary and nonbinary youth has mixed results. One recent study compared differences in mental health symptomatology and treatment seeking between nonbinary and binary youth, finding that gender binary individuals are more likely to report lower life satisfaction than nonbinary individuals.^1,18^ However, another study of Canadian youth found that nonbinary youth were more likely than binary youth to report nonsuicidal self-injury, to experience barriers when seeking medical gender affirmation, and to forego needed medical care.^19^

In the current analysis, we build on previous studies by comparing the demographic characteristics and health profiles of nonbinary and binary transgender youth recruited online for an ongoing randomized trial of a home-HIV testing intervention. The current analysis is an examination of data from the baseline survey. The analysis aims to examine whether, when recruited through social media, the profiles of binary and nonbinary youth vary. This information may be important not only for understanding more nuanced differences by gender identity, but to inform methodologies and content of research and interventions aimed at nonbinary youth.

## Methods

Data for the current analysis come from the baseline survey of Project Moxie, a randomized trial that tests the pairing of a HIPPA-secure online video counseling intervention with home HIV testing for transgender youth.^20^ Participants completed a baseline survey before being randomized into two study arms: (1) the control arm, in which participants were sent an OraQuick In-Home HIV Test^®^ and asked to report their results via an online study portal; or (2) the intervention arm, in which participants were sent an OraQuick In-Home HIV Test and asked to test while participating in motivational interviewing based counseling, testing, and referral (MI/CTR) session conducted using VSee video-calling software. The study protocol was approved by the University of Michigan Institutional Review Board and a detailed study protocol is available elsewhere.^20^

Transgender youth were recruited between June 2017 and June 2018, using advertisements and postings on social media: Facebook, Instagram, Twitter, Tumblr, and Craigslist. Recruitment materials displayed photos of transgender and gender variant persons, and included information on the $150 participation incentive. The advertisements did not mention the nature of the study, so as to avoid selection biases associated with over sampling of those with an interest in HIV or sexual health.

Advertisements linked individuals to the consent page; those who consented were directed to an eligibility screener. Six criteria were used to assess eligibility: (1) self-identification as noncisgender, indicated by a reported gender identity different from their sex assigned at birth; (2) 15–24 years old; (3) negative or unknown HIV status; (4) United States residency; (5) willingness to receive a home HIV test; and (6) access to a computer, smartphone, or tablet that supports VSee video-calling software.

A total of 698 (51.1% of those who started the screener) met the eligibility criteria for Project Moxie. Individuals were ineligible if they identified as cisgender/non-transgender (275; 20.1%), were not 15–24 years old (303; 22.2%), reported currently living with HIV (12; 0.9%), unwilling to receive a home HIV test (61; 4.5%), and did not have access to a computer, smartphone, or tablet (7; 0.5%). Of these, 329 (47.1%) created an account on the study website. An online information aggregator, Spokeo, was used to identify and delete fraudulent accounts (*n*=120, 36.5%) with a duplicate/inconsistent name, IP address outside the United States, physical address, email, or phone number. If fraudulence could not be determined, participants were contacted by study staff to verify their information.

A total of 202 individuals took the baseline survey via the study website. A two-step method was used to identify transgender youth. In the screening process, we asked sex assigned at birth (male or female). In the baseline survey, participants were asked “What is your current gender identity?” and given seven options to choose from: (1) male, (2) female, (3) trans male/trans man/trans masculine, (4) trans female/trans woman/trans feminine, (5) genderqueer/gender nonconforming, (6) agender/genderfluid, and (7) an option to write in a gender not listed. For the purpose of this analysis, those who reported a gender identity that is male, female, trans male/trans man/trans masculine or trans female/trans woman/trans feminine are categorized as gender-binary. All other reports of gender identity are categorized as gender nonbinary.

The analysis first examines differences in demographic characteristics, recent sexual health behaviors, and recent alcohol, tobacco, and other drug (ATOD) use between nonbinary and binary youth.

Second, we examined the differences in scores of nonbinary and binary youth on two subscales of the Gender Minority Stress and Resilience (GMSR) scale that were included in the Project Moxie baseline survey: community connectedness (GMSR-CC) and pride (GMSR-P).^21^ The GMSR-CC subscale consists of five items that assess degree of community connectedness using a 5-point Likert response (Strongly Disagree to Strongly Agree), and was completed by 187 participants (187/202; 92.6%). Lower scores indicate greater community connectedness. The GMSR-P subscale consists of eight items that assess degree of pride, or self-admiration. The GMSR-P subscale was adapted to consist of seven items omitting the use of the item “I am like other people but I am also special because my gender identity is different from my sex assigned at birth,” due to survey programming error. Lower scores indicate greater pride.

Finally, we used a 10-item scale developed by Garofalo et al. to measure stress among young transgender women.^15^ The scale items included problems with getting food, problems with transportation, and others on a frequency scale of 1 (“Never”) to 4 (“Often”). The life stress measure consisted of the sum of item values, with higher scores indicating greater stress exposure.

Demographic characteristics, sexual behaviors, and ATOD were compared between nonbinary and binary youth using chi-square tests of homogeneity, using a statistical significance level (α) of 0.05. Fisher's exact tests were used when the expected observations predicted on the null hypothesis of no association for any particular group were less than five. Scores on the GMSR-CC and GMSR-P scale were compared for nonbinary and binary youth using Kruskal–Wallis tests. To examine factors associated with reporting a nonbinary identity, a multivariable logistic model was fit for a binary outcome coded one if the respondent reported a nonbinary identity and zero if the participant reported a binary identity. The model included demographic characteristics, recent risky behaviors, stress, community connectedness, and pride in one's gender identity as covariates. Model-wise deletion was used; effective sample size for multivariable model was *n*=169. All analyses were conducted using Stata Version 12.0 (StataCorp).

## Results

Across the study sample, irrespective of gender identity, 66.8% of participants identified as non-Hispanic White and 33.2% identified as people of color (racial/ethnic minority); 19.3% indicated that they were unemployed, and not a student; and the highest self-reported sexual orientation, at 37.6%, was queer (Table 1). As shown in Table 1, 58.9% (119) of the 202 respondents report a binary gender, while 41.1% (83) identify as a nonbinary gender. A significantly greater proportion of binary participants were assigned a female sex at birth (AFAB) compared to nonbinary participants (82.3% AFAB vs. 67.5% AFAB, *p*=0.014) (Table 1). Youth differed significantly on medical gender affirmation, with binary youth more likely to have already accessed some medical interventions to affirm their gender (46.2% vs. 8.4%) or more likely to plan to do so in the future (49.6% vs. 43.4%), and less likely to have no plans to access medical gender affirmation (4.2% vs. 48.2%). Binary youth were more likely to be living currently as the gender that most affirms them (91.6% vs. 80.7%). There were no significant differences between binary and nonbinary participants on race/ethnicity, sexual orientation, age, education, employment, or lifetime homelessness.

**Table 1.** Demographic Characteristics of 202 Transgender Youth, United States, June 2017 to June 2018

	Total (*n*=202)	Gender		
		Binary^a^ (*n*=119)	Nonbinary^b^ (*n*=83)	
Characteristics	% (*n*)			*p*^c^
Sex assigned at birth				**0.014**
Male	23.8 (48)	17.65 (21)	32.5 (27)	
Female	76.2 (154)	82.3 (98)	67.5 (56)	
Sexual orientation				0.153
Homosexual/gay	14.4 (29)	16.0 (19)	12.0 (10)	
Bisexual	23.8 (48)	26.1 (31)	20.5 (48)	
Queer	37.6 (76)	31.1 (37)	47.0 (39)	
Other^d^	24.3 (49)	26.8 (32)	20.5 (17)	
Race/ethnicity				0.872
Non-Hispanic White	66.8 (135)	66.4 (79)	67.5 (56)	
Other race/ethnicity^e^	33.2 (67)	33.6 (40)	32.5 (27)	
Age				0.594
15–17	32.67 (66)	32.8 (39)	32.5 (27)	
18–21	46.5 (94)	48.7 (58)	43.4 (36)	
22–24	20.8 (42)	18.5 (22)	24.1 (20)	
Has graduated high school (includes GED)				0.812
Yes	70.8 (143)	71.4 (85)	69.9 (58)	
No	29.2 (59)	28.6 (117)	132.1 (25)	
Employment status				0.463
Unemployed, not a student	19.3 (39)	21.0 (25)	16.9 (14)	
Employed and/or student	80.7 (163)	79.0 (94)	83.1 (69)	
Ever been homeless				
Yes	19.3 (39)	18.5 (22)	20.5 (17)	0.724
No	80.7 (163)	81.5 (97)	79.5 (66)	
Accessed any medical interventions to affirm their gender				**<0.001**
Yes	30.7 (62)	46.2 (55)	8.4 (7)	
No, but I plan to	47.0 (95)	49.6 (59)	43.4 (36)	
No, and I do not plan to	22.3 (45)	4.2 (5)	48.2 (40)	
Currently living as the gender that most affirms them				**0.023**
Yes	87.1 (176)	91.6 (109)	80.7 (67)	
No	12.9 (26)	8.4 (10)	19.3 (16)	

Bold values indicate significant results (*p*<0.05).

^a^ Includes 82 trans man/trans masculine/trans male, and 37 trans woman/trans feminine/trans female.

^b^ Includes 50 Genderqueer/Gender nonconforming, 25 Agender/Genderfluid, 7 nonbinary, and 1 two-spirit male.

^c^ Results from Chi-square tests of homogeneity.

^d^ Includes 18 pansexual, 14 questioning/unsure, 9 heterosexual/straight, 3 asexual, 2 demisexual, 1 polysexual, and 1 sexually fluid.

^e^ Includes 16 Hispanic, 12 Black, 7 Asian, 3 Middle Eastern, 2 Native American/Alaskan Native, and 27 multiracial individuals.

GED, general educational development.

Nonbinary and binary youth were similar across most measures of stress, resilience, and health risk, with the exception of life stress, with nonbinary youth reporting higher median stress levels (19 vs. 16 on a scale from 10 to 40, *p*=0.007) (Table 2). Community connectedness and pride did not vary significantly between nonbinary and binary youth (*p*=0.914; *p*=0.234), nor did indicators of sexual behavior and ATOD.

**Table 2.** Measures of Stress, Resilience, and Health Risks in 202 Binary and Nonbinary Transgender Youth, United States, June 2017 to June 2018

		Total	Gender		*p*^a^
			Binary	Nonbinary	
Resilience and stress factors	Minimum–maximum		Mean, median		
Community connectedness^b^	5–25	***n*=187**	12.5, 13	12.4, 12	0.914
Pride^c^	7–35	***n*=188**	19.5, 20	20.8, 20	0.234
Stress^d^	10–40	***n*=183**	17.4, 16	19.9, 19	**0.007**

Risk factors		% (*n*)			*P*^e^
Sexual behaviors					
Overall		***n*=202**	***n*=119**	***n*=83**	
No. of sexual partners in the past 3 months					0.753
0		29.7 (60)	27.7 (33)	32.5 (27)	
1		42.1 (85)	42.9 (51)	41.0 (34)	
≥2		28.2 (57)	29.4 (35)	26.5 (22)	
Subsets reporting at least one sex partner		***n*=142**	***n*=86**	***n*=56**	
No. of partners with who engaged in condomless anal sex		***n*=54**	***n*=33**	***n*=21**	0.274^f^
0		25.9 (14)	18.2 (6)	38.1 (10)	
1		50.0 (27)	54.5 (18)	42.9 (9)	
≥2		24.1 (13)	27.3 (9)	19.0 (4)	
No. of partners with who engaged in condomless vaginal sex		***n*=90**	***n*=51**	***n*=39**	1.000
0		58.9 (53)	58.8 (30)	59.0 (53)	
1		32.2 (29)	31.4 (16)	33.3 (13)	
≥2		8.9 (8)	9.8 (5)	7.7 (3)	
ATOD use in the past 90 days					
		***n*=202**	***n*=119**	***n*=83**	
Alcohol					0.560
Yes		68.8 (139)	67.2 (80)	71.1 (59)	
No		31.2 (63)	32.8 (39)	28.9 (24)	
		***n*=120**	***n*=68**	***n*=52**	
Underage alcohol use					0.571
Yes		78.3 (94)	76.5 (52)	80.8 (42)	
No		21.7 (26)	23.5 (16)	19.2 (10)	
		***n*=202**	***n*=119**	***n*=83**	
Tobacco					0.371
Yes		36.1 (73)	33.6 (40)	39.8 (33)	
No		63.9 (129)	66.4 (79)	60.2 (50)	
Marijuana		***n*=187**	***n*=112**	***n*=75**	0.444
Yes		51.0 (103)	48.7 (58)	54.2 (45)	
No		49.0 (99)	51.3 (61)	45.8 (38)	
Other drugs^g^					0.547
Yes		20.9 (39)	22.3 (25)	18.7 (14)	
No		79.1 (148)	77.7 (87)	81.3 (61)	

Bold values indicate significant results (*p*<0.05).

^a^ Results from Kruskal–Wallis equality-of-populations rank tests.

^b^ Low values indicate more connectedness.

^c^ Low values indicate more self-admiration.

^d^ High values indicate more stress.

^e^ Results from Chi-square tests of homogeneity.

^f^ Results from Fisher's exact tests.

^g^ Includes synthetic marijuana, cocaine, amphetamine type stimulants, amyl nitrates, sedatives, hallucinogens, and opioids.

ATOD, alcohol, tobacco, and other drug.

Across the sample, 28.2% (57/202) of transgender youth reported having two or more sexual partners in the past 90 days. Of those reporting at least one sexual partner in the past 90 days, 74.1% (40/54) engaged in condomless anal sex at least once, and 41.1% (37/90) engaged in condomless vaginal sex at least once. Alcohol use across the sample in the past 90 days was 68.8% (139/202), while alcohol use among transgender youth who were under the legal drinking age of 21 was 78.3% (94/120). Tobacco, marijuana, and other drug use in the past 90 days was 36.1% (73/202); 51% (103/187); 20.9% (39/187), respectively. The reporting of these behaviors did not vary significantly by gender identity.

Table 3 displays factors independently associated with reporting a nonbinary identity compared to a binary identity. Relative to those assigned male at birth (AMAB), those AFAB were less likely to report a nonbinary identity (odds ratio [OR] 0.353, 95% confidence interval [CI] 0.136–0.915). Those who had accessed, or planned to access, medical gender affirmation were less likely to report a nonbinary identity than those who had not, or did not intend to seek medical affirmation (OR 0.037, 95% CI 0.011–0.124). Pride in one's gender identity was more likely to be associated with a binary identity (OR 1.064, 95% CI 1.005–1.127), while high levels of stress were more likely to be associated with a nonbinary identity (OR 1.072, 95% CI 1.010–1.138).

**Table 3.** Logistic Regression Analysis for Factors Associated with Nonbinary Gender Identity in Transgender Youth (*n*=169), United States, June 2017 to June 2018

	OR	CI	*p*
Characteristics			
Sex (AMAB)^a^	0.353	0.136–0.915	0.032
Race (White)^b^	0.586	0.242–1.405	0.229
Age (15–17)^c^			
Age (18–21	0.687	0.290–1.631	0.395
Age (22–24)	0.537	0.171–1.681	0.285
Currently living as the gender that most affirms them	0.873	0.283–2.691	0.813
Accessed or planned to access any medical affirmation	0.037	0.011–0.124	<0.001
Stress and resilience			
Community connectedness	0.932	0.854–1.018	0.116
Pride	1.064	1.005–1.127	0.033
Life stress	1.072	1.010–1.138	0.022

^a^ Sex reference category: male.

^b^ Race reference category: White.

^c^ Age reference category: 15–17 years old.

AMAB, assigned male at birth; CI, confidence interval; OR, odds ratio.

## Discussion

These data add to the nascent literature on the health and demographic profiles of nonbinary adolescents, highlighting areas of similarity and key differences between nonbinary and binary youth. Striking differences emerged between nonbinary and binary youth around medical gender affirmation, potentially indicative of disparities in access to gender-affirming medical care for nonbinary youth. Although binary youth are more likely to desire medical intervention than nonbinary youth, over half of nonbinary youth in our sample desired or had already undergone medical gender affirmation, challenging traditional narratives that only binary transgender individuals desire endocrine therapy or other medical interventions.^22–24^

Nonbinary youth who desired medical gender affirmation were far less likely to have actually obtained such services, with a 5:1 ratio of nonbinary youth who wanted to access gender-affirming care compared to those who had done so (43.4% vs. 8.4%), as compared to a ratio of ∼1:1 among binary youth (49.6% vs. 46.2%). While some of these nonbinary youth may be choosing to delay medical affirmation, this finding is in line with other studies that have found that nonbinary individuals have greater barriers to accessing gender-affirming care.^6,19^ In one Canadian study, nonbinary youth were less likely to have a primary care doctor and, among those who had one, less likely to be out to their provider.^19^ There may also be disparities for nonbinary youth in parental support for medical transition.

Lastly, health care providers may be uncomfortable with nonbinary genders and so-called “partial treatment,” that is, some medical intervention that is not as extensive as what would typically be used to hormonally and surgically transition to the “opposite sex.”^25^

Older versions of the World Professional Association for Transgender Health Standards of Care emphasized “complete” transition to the “opposite” sex, and laid out a “gatekeeping” model where a mental health professional had to diagnose the individual with gender identity disorder and support their use of medical affirmation services before the patient could access them.^26,27^ The patient also had to undergo an extended “real life experience” in the “opposite” sex before permission for medical affirmation services could be obtained. Research indicates that this model does not work for all, especially nonbinary youth, who may need more flexible plans.^25^

With increasing evidence that gender-affirming procedures can lower levels of depression, anxiety, and stress in transgender individuals, more relevant and nuanced approaches to gender affirmation care for nonbinary youth are needed.^6,19,28,29^ The newer informed consent model, practiced by many medical centers and clinics, puts the power of accessing gender-affirming care in the hands of the clients, rather than medical professionals who may not understand the needs of this population.^27^

Levels of community connectedness were low for all transgender youth. As nonbinary youth reported higher levels of stress than their binary counterparts, lower levels of resilience are especially concerning, given that the literature finds a sense of pride and community as related to being able to mediate stress, and can deter engagement in risky health behaviors.^30,31^ Recent studies have examined connectedness among transgender populations, and identified a relationship between belongingness, security in one's identity, and well-being.^32^

Although nonbinary and binary youth were similar across many demographic dimensions and health behaviors there were several key differences. Binary respondents in the current study were more likely than nonbinary respondents to indicate being AFAB, contrary to recent literature where nonbinary individuals have been more likely to indicate being AFAB.^19,33^ We suggest that our marketing strategy and method of engagement with youth may have felt more inclusive to nonbinary AMAB youth. Recruitment materials included depictions of young people with a variety of gender expressions to encourage inclusivity and the participation of transgender youth with a variety of genders. It is possible that AMAB youth may have felt more represented and free to report a nonbinary gender identity within an expansive list of genders, including a write-in option.

Across the sample, transgender youth exhibited high levels of alcohol, tobacco, and marijuana use. The Youth Risk Behavior Surveillance Survey, conducted by the Centers for Disease Control and Prevention, provides data representative of high school students in the United States with 29.8% reporting current alcohol use and 19.8% reporting current marijuana use.^34^ In the current study, the levels of ATOD use reported among transgender youth were significantly higher: 36.1% of transgender youth reported alcohol use, while 51.0% reported marijuana use, in the past 90 days. Such high levels among transgender youth could illustrate the negative impacts of high stress and social stigma.

### Limitations

As language in the transgender community is continually expanding, it is likely that the options given to report gender identity failed to account for youth of other genders. Moreover, survey respondents were coded as binary if they selected trans man/trans masculine/trans male or trans woman/trans feminine/trans female; youth did not directly identify as binary or nonbinary, introducing the possibility of misclassification bias. It is possible some trans masculine and trans feminine individuals, for instance, identify their gender as nonbinary as well. Additionally, these data represent only those who had access to computers and the online recruitment strategies used, which may have left out rural and/or low-income youth, and transgender youth experiencing homelessness.

Furthermore, while the response rate for completed survey items was 92.6%, of the 329 who met the eligibility criteria, 56.8% completed the survey. Finally, the small sample size of transgender youth of color precluded an exploration of understanding of how the experiences of binary and nonbinary youth compared across racial and ethnic identity, a significant limitation given the potentially unique experiences of Black, Latinx, Middle-Eastern, indigenous, multiracial, and other transgender youth of color. The sample of survey respondents was nonrepresentative of transgender individuals in other ways, such as being comprised of three-quarters AFAB individuals, limiting data on AMAB individuals.

## Conclusion

The comparison of the demographic and behavioral profiles of gender binary and nonbinary youth illustrates the need for data specific to nonbinary youth to be included in research efforts. Allowing for a full range of gender identities to be expressed when recording identity in surveys should be considered the gold standard for identity data collection.

Low levels of resilience among nonbinary youth, coupled with high reported levels of stress and associated high levels of ATOD use, suggest that nonbinary youth are experiencing many of the same problems that have been well-documented for binary youth, yet there is currently a lack of adaptable health care and social support that is tailored to their unique needs. These findings point to a need for refinement of the transgender clinical care model, especially concepts around linear transition and goal of presenting as the “opposite” sex, which further marginalizes nonbinary youth. Training health care practitioners to more accurately assess the needs of nonbinary youth and use more inclusive care models is critical to decreasing stress.

Interventions need to take the nuanced needs of nonbinary individuals into account, which may involve hiring nonbinary research team members, using consultants, and/or forming community advisory boards to ensure projects are inclusive of all members of the transgender population.^35^ Future research is needed to understand how socialization and sex assigned at birth may contribute to risk behaviors in transgender youth of all gender identities, and further explore the health profiles of nonbinary youth. Additionally, future research is needed to understand the broader social influences on the health of nonbinary youth. Especially of youth who may still live at home, and/or are of school-age. Nonbinary youth already experience high stress and social stigma; research and interventions should work to ameliorate these concerns, and avoid data collection, research, and intervention processes that may unintentionally further stigmatize nonbinary youth.

## Disclaimer

The content of this publication is solely the responsibility of the authors and does not necessarily represent the official views of the National Institutes of Health.

## Abbreviations Used

AFAB: assigned a female sex at birth
AMAB: assigned male at birth
ATOD: alcohol, tobacco, and other drug
CI: confidence interval
GMSR-CC: Gender Minority Stress and Resilience community connectedness
GMSR-P: Gender Minority Stress and Resilience pride
OR: odds ratio
